# Relating Radical Delocalization, Charge Transfer, and Magnetic Ground State in Acene-Derived Oxyradicals

**DOI:** 10.1021/acs.nanolett.5c00263

**Published:** 2025-04-14

**Authors:** Tao Wang, Sergio Salaverría, Fernando Aguilar-Galindo, Javier Besteiro-Sáez, Luis M. Mateo, Paula Angulo-Portugal, Jonathan Rodríguez-Fernández, Dolores Pérez, Martina Corso, Diego Peña, Dimas G. de Oteyza

**Affiliations:** ‡ State Key Laboratory of Organometallic Chemistry, Shanghai Institute of Organic Chemistry, University of Chinese Academy of Sciences, Chinese Academy of Sciences, Shanghai 200032, China; § Donostia International Physics Center, 20018 San Sebastián, Spain; ⊥ Nanomaterials and Nanotechnology Research Center (CINN), CSIC-UNIOVI-PA, 33940 El Entrego, Spain; ∥ Departamento de Química, Universidad Autónoma de Madrid, 28049 Madrid, Spain; ¶ Institute for Advanced Research in Chemical Sciences, (IAdChem), Universidad Autónoma de Madrid, 28049 Madrid, Spain; # Centro Singular de Investigación en Química Biolóxica e Materiais Moleculares (CiQUS) and Departamento de Química Orgánica, Universidade de Santiago de Compostela, 15782 Santiago de Compostela, Spain; ∇ Centro de Fisica de Materiales (CFM/MPC), CSIC-UPV/EHU, 20018 San Sebastián, Spain; ○ Physics Department, University of Oviedo, 33007 Oviedo, Spain; ◆ Oportunius, Galician Innovation Agency (GAIN), 15702 Santiago de Compostela, Spain

**Keywords:** carbon nanostructures, π-magnetism, open-shell, charge transfer, on-surface synthesis

## Abstract

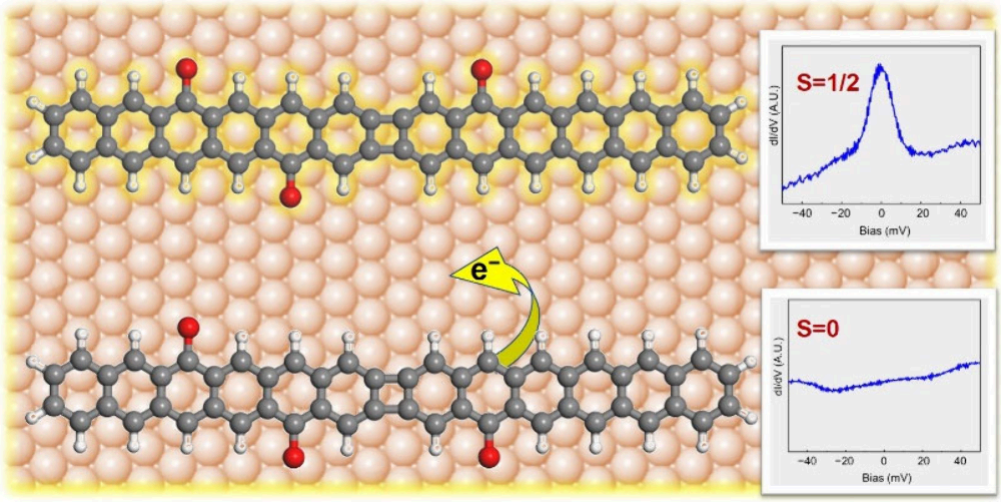

At the same time that our capabilities to synthesize open-shell carbon-based materials are rapidly growing with the development of on-surface synthesis under vacuum conditions, interest in π-magnetism is rising due to its excellent prospects for potential applications. As a result, increasing efforts are being focused on the detailed understanding of open-shell carbon nanostructures and all of the parameters that determine their spin densities and magnetic ground states. Here we present a facile route to synthesize different open-shell acene derivatives with closely related structures by the addition of functional groups. A systematic comparison allows us to draw conclusions on the role of the functional groups and their number and distribution, as well as on the role of the radical state delocalization in relation with the presence or absence of charge transfer at interfaces, which consequently affects the molecule’s π-magnetism.

The development of on-surface synthesis,^1,2^ a variant of organic chemistry commonly performed under vacuum conditions, has opened new doors for the synthesis and characterization of open-shell molecules that would otherwise be unstable in conventional solution-phase chemistry.^3−7^ In addition, the large spin delocalization and the low spin–orbit coupling associated with carbon-based materials appear as potentially relevant advantages for applications when compared to conventional magnetic systems relying on d or f states of transition metals.^8−11^ Therefore, increasing research efforts are being focused on the development and understanding of carbon-based materials displaying π-magnetism.^3−5,12^

Several electronic descriptors contribute to define the presence (or absence) of magnetism in carbon nanostructures, as well as their magnetic ground state.^3−5,12^ Importantly, some of these parameters often depend on one another, making an understanding of the interrelations instrumental for the rational design of optimized molecular structures. Two key parameters are (i) the energy *E*_0_ of the molecular orbitals close to the Fermi level and (ii) the Coulomb repulsion *U* felt by the electrons occupying those low energy orbitals.^13^ Large *U* values may prevent the double occupancy of an electronic state, which is the basis for the presence of unbalanced spin densities and magnetic moments. That is, if a state of energy *E*_0_ is populated with a spin-up electron, the spin-down electron will lie at an energy *E*_0_ + *U*, which may be above the Fermi level and thereby prevent its occupancy (Figure 1a). Taking into consideration the state width Δ, which increases with stronger molecule–substrate hybridization, the orbital’s tails may cross the Fermi level, modifying the spin-up and spin-down occupancies from 1 and 0 to ρ_up_ and ρ_down_. Such partial occupancies in turn renormalize the energies for both the spin-up and spin-down electrons to *E*_0_ + ρ_down_*U* and *E*_0_ + ρ_up_*U*, respectively (Figure 1a). From this model, one can already infer that the ionization energy *E*_0_, the Coulomb repulsion *U*, and the state width Δ jointly affect the resulting spin density (ρ_up_ – ρ_down_), which is maximized by large *U*, small Δ, and *E*_0_ ≈ −*U*/2.^13^

**Figure 1:**
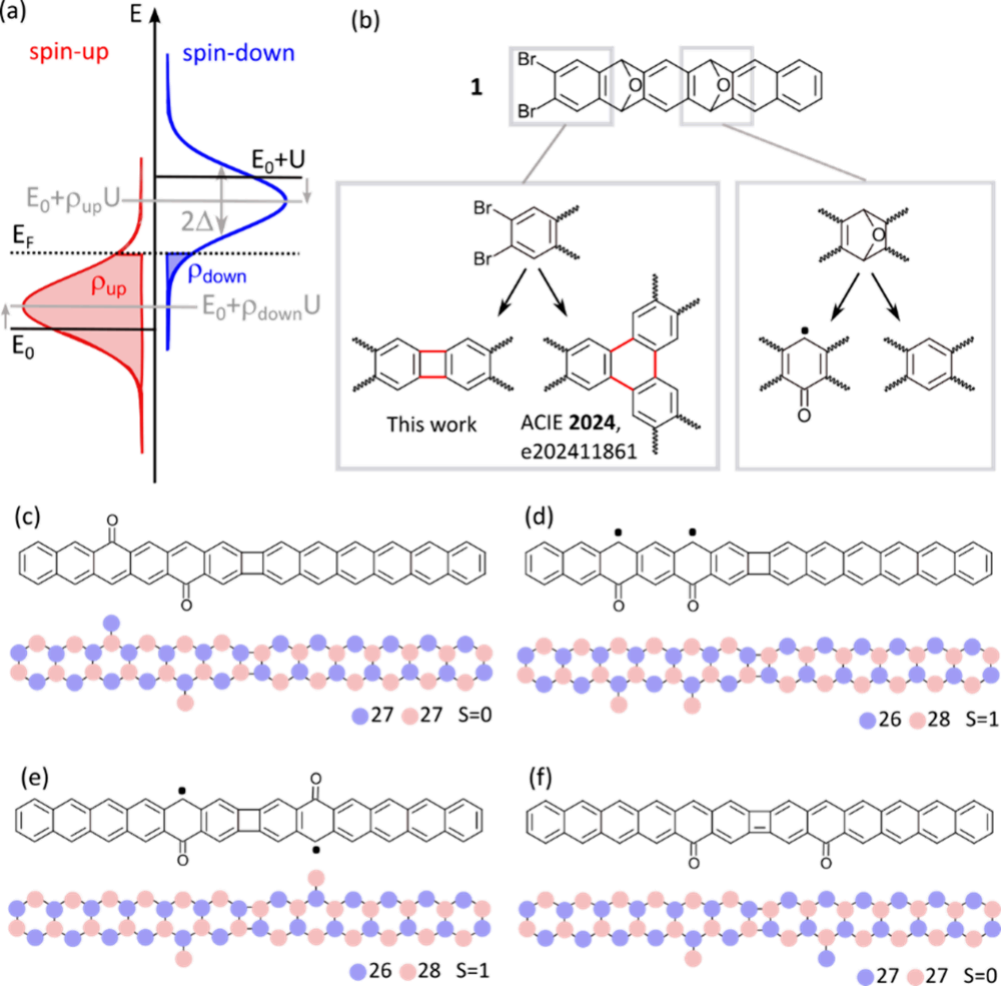
(a) Schematic picture of the Anderson model, according to which a magnetic state with energy *E*_0_ is populated by a spin-up electron, whereas the virtual state for the spin-down electron lies at *E*_0_ + *U*, above the Fermi level. As the states have a width Δ, their tails may cross the Fermi level, leading to noninteger occupancies ρ_up_ and ρ_down_ that further renormalize the Coulomb gap. (b) Precursor molecule **1**, featuring two types of functionalization with (i) halogens (left) and (ii) epoxy groups (right). Upon annealing, the former leads to intermolecular coupling through bonds as marked in red, whereas the latter either transforms into carboxy groups generating an associated radical or comes off entirely. (c–f) Schematic representation of cyclobutadiene-containing hexacene dimer derivatives featuring two carbonyl groups at varying positions. The associated open- or closed-shell nature of the products is revealed with Kekulé structures, as well as with Ovchinnikov’s rule or Lieb’s theorem for bipartite structures, according to which the magnetic ground state of molecules directly depends on the number of atomic sites of each atomic sublattice (here pictured in red and blue).

To explore interrelations between the different electronic descriptors and the molecules’ open-shell character, in this work we characterize a model molecular family consisting of linear hexacene dimers with carbonyl group functionalization, linked through central four-membered rings. Cyclobutadiene-containing acene analogues partially disrupt the conjugation along the linear polycyclic system and have attracted much attention, with numerous studies both in solution^14−17^ and on surfaces under vacuum.^18−24^ By fine-tuning the chemical structure within this family, we observe a varying magnetic ground state that we can explain with ionization energy variations (that is, *E*_0_ variations in Figure 1a) inversely correlated with the spatial extent of the electronic states.

The detailed molecular precursor structure is pictured in Figure 1b as **1**, consisting of an ortho-dibrominated hexacene derivative further functionalized with two epoxy groups to increase its solubility and stability under ambient conditions. Ortho-dihalogenated derivatives have been previously used for the surface-supported synthesis of a variety of dimer and trimer structures through [2 + 2] and [2 + 2 + 2] cycloaddition reactions, respectively (Figure 1b).^18,20−22,25^ Particularly focusing on compound **1**, its [2 + 2 + 2] trimerization has served for the on-surface synthesis and characterization of [19]starphene.^25^ However, its most commonly followed reaction path on Au(111) is the [2 + 2] cycloaddition that results in the formation of linear dimers (Figure S1), which can be considered as phenylene analogues to tridecacene derivatives.^23^

As readily described for the synthesis of [19]starphene,^25^ the epoxy groups most commonly transform into carbonyl groups upon annealing. This transformation is probably initiated by the C–H cleavage on a bridgehead carbon atom of an epoxy group, followed by a C–O cleavage to form a carbonyl derivative (Figure S2a). Transforming an epoxy into a carbonyl group drives the rehybridization of the involved carbon atoms from sp^3^ to sp^2^, at the same time as the oxygen atom contributes with one extra electron from a p_*z*_ orbital to the π-electron network.^26^ As a result, an odd number of p_*z*_ electrons is added to the system, which causes the generation of a π-radical as pictured in Figure 1b, also called an oxyradical.^27^ However, a fraction of the epoxy groups that vary with the annealing treatment (Figure S3) also dissociate entirely (Figure 1b). This reaction is based on two successive C–O cleavages on the epoxy group to form the corresponding acene (Figure S2b).

The molecules under study can be described as nanographenes and therefore as composed by a bipartite lattice (that is, the atoms of each lattice only bind to the atoms of the opposite lattice, shown in red and blue, respectively, in Figure 1c–f). This even holds for the dimers, although the four-membered ring causes a switching of the lattices from one acene side to another (Figure 1c–f).^28,29^ Therefore, if two carbonyl groups functionalize the same hexacene fragment but on opposite sides, the radicals locate on opposite sublattices and will quench each other, forming a π-bond (Figure 1c). Instead, if the carbonyls are on the same hexacene segment side, the radicals locate on the same sublattice and can no longer quench each other (Figure 1d). As expected, Ovchinnikov’s rule^30^ and Lieb’s theorem^31^ support this scenario and predict their ferromagnetic alignment with a *S* = 1 ground state (Figure 1d). If the two radicals are separated by a four-membered ring, the opposite scenario applies. Carbonyl groups on the same side allow for the radicals to quench into a closed-shell structure (Figure 1f), whereas carbonyl groups on opposite sides cause the generation of two radicals with ferromagnetically aligned spin moments (Figure 1e).

An archetypical reaction product after deposition of **1** onto a Au(111) surface preheated to 230 °C is dimer **2** in Figure 2, in which the four epoxy groups transform into carbonyl groups that decorate alternating sides of each of the hexacene segments. The four radicals generated by the carbonyl groups quench pairwise and consequently this is a closed-shell molecule. However, products in which some of the epoxy groups entirely dissociate are also present. Out of the manifold of products obtained, this is exemplified with the selected structures **3** and **4** (featuring three carbonyl groups), **5** (two carbonyl groups), and **6** (only one carbonyl group). From the arguments outlined above, in their charge-neutral state, structures **3**, **4**, and **6** have an odd number of p_*z*_ electrons and therefore must be open-shell molecules, as they cannot pair up all of their electrons. Structure **5** has an even number of p_*z*_ electrons. However, the two carbonyl groups generate radicals on the same sublattice and can therefore not quench each other, but instead align ferromagnetically.

**Figure 2:**
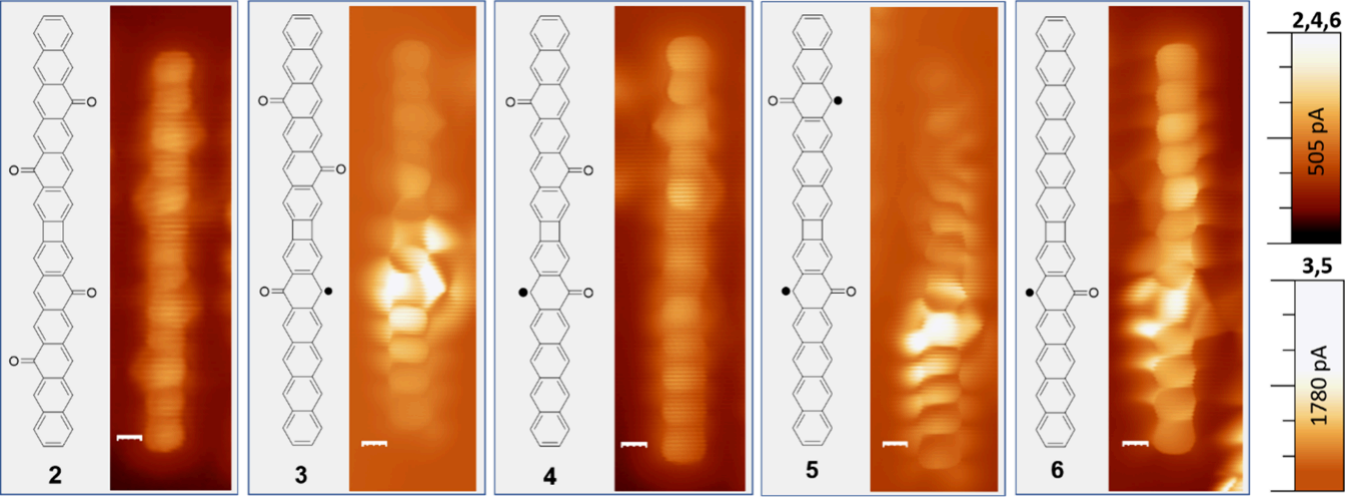
Wireframe chemical structure drawing for selected dimer products named **2**–**6**, with decreasing number of carbonyl groups, in a charge-neutral state. Next to them appear their associated bond-resolving STM images as acquired on Au(111). Scanning parameters for all bond-resolving STM images at constant height mode: 5 mV. Scale bar: 2 Å. Common color scales for molecules **2**, **4**, and **6** and for molecules **3** and **5** are shown on the right.

To probe the open-shell nature of the molecules and assess the exchange interactions proposed across four-membered rings,^28^ we performed low-energy scanning tunneling spectroscopy experiments.^3^ The carbonyl groups promote relatively strong intermolecular interactions via hydrogen bonds that drive molecular clustering (Figures S1 and S3). The characterization has been performed on the aggregated molecules, with reproducible results independently of the cluster size or of the exact structures of neighboring molecules. Representative spectra acquired on each of these molecules are shown in Figure 3, vertically shifted with respect to one another for the sake of clarity. Ovchinnikov’s rule^30^ predicts a ground state *S* = 1/2for **3**, **4**, and **6** and *S* = 1 for **5**. The expected spectral shape would thus be a strong Kondo resonance for the three former and a weaker zero bias resonance for the latter, associated with an underscreened Kondo effect.^32−34^ This, however, does not fit the experimental curves displayed in Figure 3. The absence of inelastic excitations or Kondo peaks hints at a closed-shell nature for **4** and **6**, whereas the zero bias resonances with Frota function line shape and full-width at half-maximum (fwhm) of 7.2 and 7.8 mV for **3** and **5**, respectively, rather support a *S* = 1/2 ground state and its associated Kondo resonance.^26^

**Figure 3:**
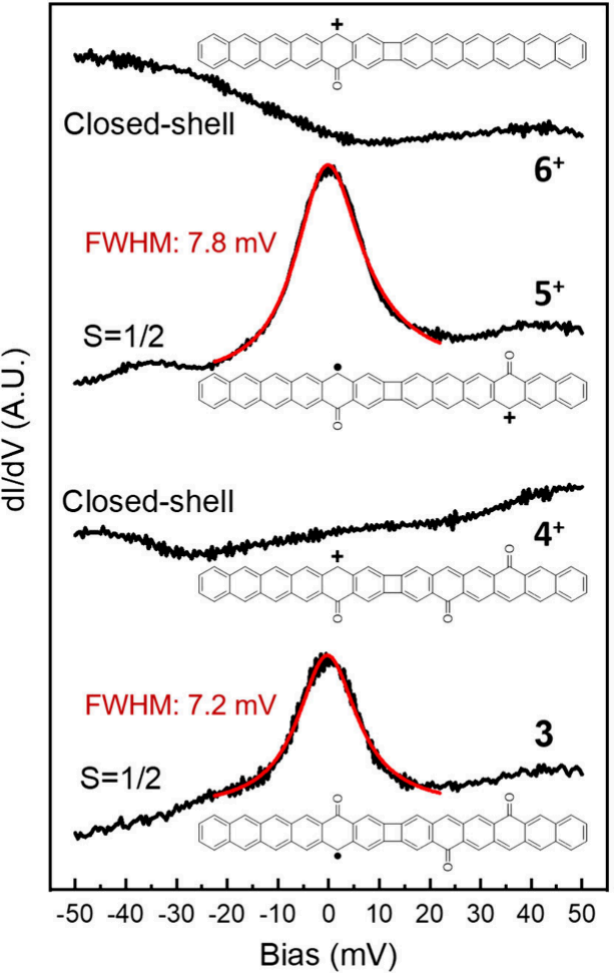
Representative differential conductance point spectra acquired on molecules **3**, **4**, **5**, and **6**, where the latter three turn out to be positively charged on Au(111). Frota function fits are superimposed on the spectra of **3** and **5**, revealing fwhm of 7.2 and 7.8 mV, respectively. Lock-in amplitude/frequency 2 mV/731 Hz.

The experimental measurements can be reconciled with the predictions by considering that charge transfer between molecules and the metallic substrate can occur. The notably higher work function of Au(111) (5.3 eV),^35^ compared to graphene (4.4 eV)^36^ and graphene low-dimensional fragments or molecules, promotes the charge transfer of low energy molecular electrons to the Au substrate. The π-radicals of carbon nanostructures are low energy states and therefore particularly prone to be transferred to the underlying Au substrate, as readily observed in numerous other carbon nanostructures.^37−40^ Charge transfer of one electron to the substrate for each of the molecules would cause structures **3**^**+**^, **4**^**+**^, and **6**^**+**^ to become closed-shell, whereas **5**^**+**^ would display a net spin of *S* = 1/2. In this case, however, it is the unambiguous Kondo resonance observed on molecule **3**, which is in discrepancy with the closed-shell structure predicted for **3**^**+**^. That is, while molecules **4**, **5**, and **6** indeed become positively charged on Au(111), structure **3** behaves otherwise and remains neutral.

The same conclusion can be reached from conductance spectroscopy over a wider range, whereby we probe the energy and the spatial distribution of the density of states of molecular orbitals. Figure 4a shows conductance point spectra acquired on molecules **3**, **4**, and **5** (**6** did not provide satisfactory measurements and is therefore not included). On the basis of the combination of the point spectra with conductance maps, we assign the energies of the two highest occupied resonances and the lowest unoccupied resonance to the solid, dashed, and dotted lines marked on each spectrum, respectively. Figure 4b shows representative conductance maps associated with each of the marked resonances, along with simulated images of the two highest energy occupied molecular orbitals and of the lowest energy unoccupied molecular orbital of gas-phase molecules. An excellent fit is obtained between the experiment and theory. Good agreement, however, is only obtained for neutral **3** but positively charged **4**^**+**^ and **5**^**+**^, providing further support to our previously reached conclusion. It should be noted that for **4**^**+**^ the order of HOMO and HOMO–1 is reversed with respect to the measurements, but we assign that discrepancy to the used model (e.g., relying on gas-phase calculations that neglect effects from hybridization with the substrate).

**Figure 4:**
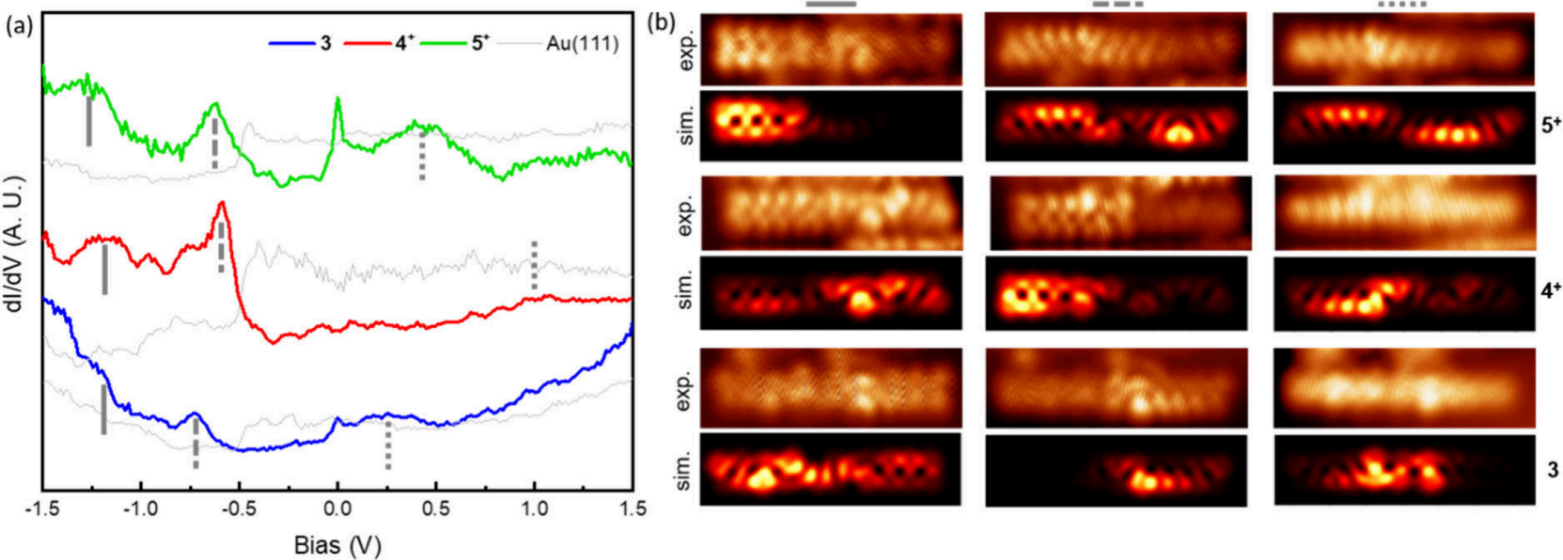
(a) Representative differential conductance point spectra acquired on molecules **3** (blue), **4** (red), and **5** (green), along with the reference spectra (thin, gray) acquired for each case on nearby Au(111). The reference energies for the two highest energy occupied molecular resonances and the lowest energy unoccupied molecular resonance are marked with solid, dashed, and dotted lines, respectively. (b) Representative conductance maps for each of the three molecular resonances on each molecule and their comparison to simulations for the two highest occupied and the lowest unoccupied molecular orbitals of **3**, **4**^**+**^, and **5**^**+**^. In the case of molecule **4**^**+**^, the order of the two calculated occupied orbitals has been switched to fit the experimental data.

The reason behind the different charge transfer of molecules **4**, **5**, and **6** compared to **3** is the following. The occurrence (or its absence) of charge transfer between a molecule and its substrate depends on the energy of the molecule’s highest occupied (or singly occupied) molecular orbital (that is, *E*_0_ in Figure 1a) relative to the substrate’s Fermi level. According to Koopman’s theorem the HOMO (or SOMO) energy corresponds to the ionization energy.^41,42^ Assuming a vacuum level alignment of molecule and substrate,^43,44^ a direct comparison of ionization energy and work function would thus determine the presence or absence of charge transfer. Unfortunately, the real picture is not as simple because additional factors need to be taken into account like, e.g., work function modifications by interface dipoles upon molecular adsorption,^43−45^ but it holds that higher ionization energies tend to prevent electron transfer from molecule to substrate, whereas it is facilitated by lower ionization energies.

The addition of electron-withdrawing groups like halogens or carbonyls to polyaromatic hydrocarbons (PAHs) drives a depletion of the electron density over the extended π-network, eventually generating so-called π-holes.^46,47^ Because frontier orbitals in PAHs are typically π-orbitals, their lowered electron density results in a stronger attraction by the nuclei and hence in increased ionization energies,^48−50^ intuitively making molecules **6** (one carbonyl) and **5** (two carbonyl groups) more prone to transferring an electron to the high work function Au(111) substrate than molecules **3** and **4** (three carbonyl groups). We have thus calculated the ionization energies of neutral molecules **3**–**6** in the gas phase by density functional theory (DFT), and the results are presented in Table 1. For the sake of completeness, we have also added molecule **2**. It is expected to show the highest ionization energy, not only due to the electron-withdrawing effect of the highest number of carbonyl groups but also due to its more stable closed-shell electronic structure.

**Table 1:** Calculated Ionization Energies for Molecules **2**–**6** in the Gas Phase

	**2**	**3**	**4**	**5**	**6**
ionization energy (eV)	6.167	5.918	5.765	5.764	5.548

The trend outlined above is qualitatively confirmed by the calculations, increasing the ionization energy with increasing number of carbonyl groups. In addition, it shows a notably larger ionization energy for **3** than for **4**. This explains the charge transfer from **4** to the Au(111) and its absence for **3**, all in all providing a fully coherent picture for the varying charge transfer and the associated magnetic ground state of the molecules under study. However, at this stage it seems unclear why these two molecules with the same number of carbonyl groups and very similar structures should have such different ionization energies.

We rationalize that difference based on the resonant forms that each molecule can acquire. Figure 4a shows the most intuitive resonant structure that may be drawn for **3**, in which one radical is created on the opposite side of each carbonyl group (Figure 1b) and the two radicals on the same hexacene segment but opposite sides (marked with a green dashed square) quench each other, as explained in Figure 1c. This leaves a single radical on the left hexacene segment. The radical drawn in Figure 5a on the carbon atom opposite of the carbonyl can be shifted to any carbon atom of the same sublattice on the same hexacene segment and all the way to the first carbonyl of the neighboring hexacene (see selected examples of resonant structures in Figure S4). However, as explained in Figure 1e, radicals on opposite sides and separated by a four-membered ring cannot quench each other (red dashed box in Figure 5b), hindering the presence of a single radical on the right hexacene segment. All of the above can be directly correlated with the molecular orbital of the singly occupied state as calculated by DFT (Figure 5c), in which one can immediately see that the orbital mainly locates on one sublattice of the left hexacene segment, extending to the first carbonyl of the neighboring hexacene segment but being completely absent thereafter.

**Figure 5:**
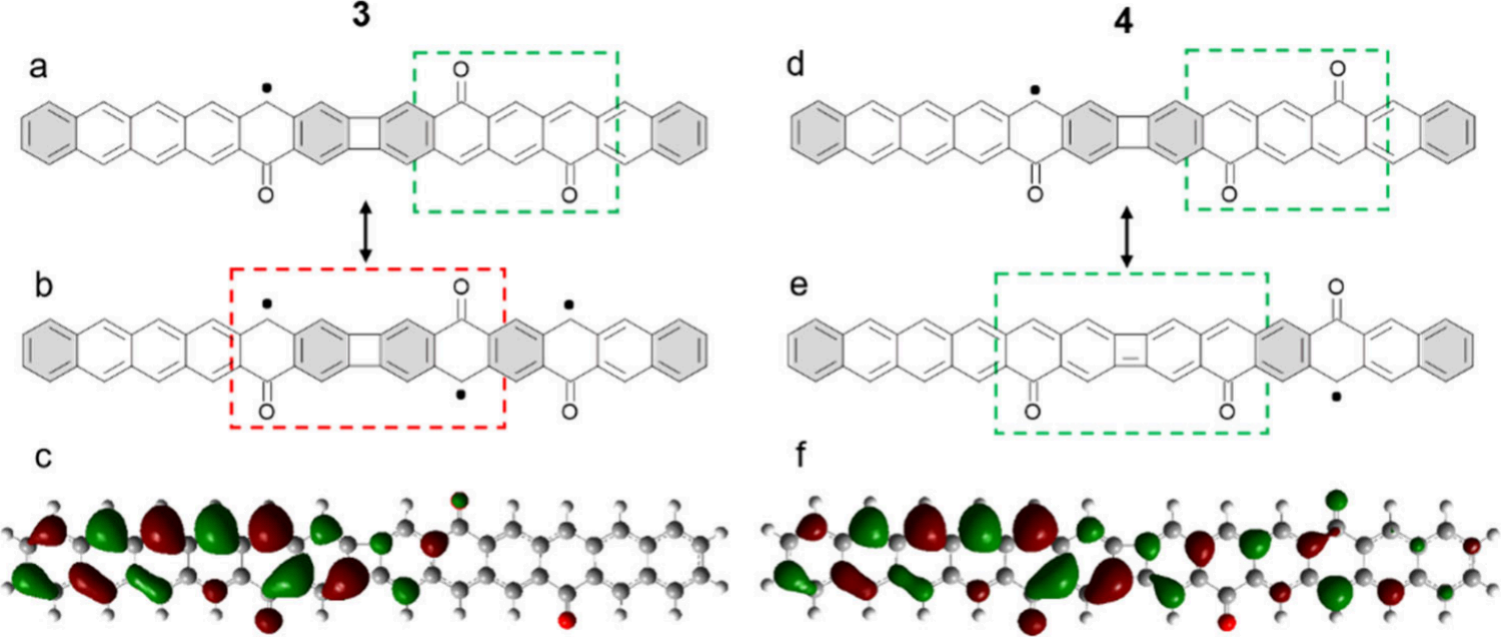
(a) Resonant structure of molecule **3** in which the radicals generated by the two carbonyl groups within the green dashed rectangle quench each other. Clar sextets are marked with a gray background. (b) Resonant structure of **3** with three radicals (and thus energetically very unfavorable) in which the two radicals within the red dashed rectangle cannot quench each other. (c) Singly occupied molecular orbital as calculated by DFT. (d) Resonant structure of molecule **4** in which the radicals generated by the two carbonyl groups within the green dashed rectangle quench each other. (e) Resonant structure of molecule **4** in which the radicals generated by the two carbonyl groups within the green dashed rectangle quench each other, leaving a single radical on the right hexacene moiety. (f) Singly occupied molecular orbital as calculated by DFT.

Looking now into structure **4**, Figure 5d shows the most intuitive resonant structure. As for **3**, the two radicals on the right hexacene segment quench each other (marked with a green dashed square and explained in Figure 1c), leaving a single radical on the left hexacene segment. However, a resonant form in which the two central radicals quench each other leaving behind a single radical on the right hexacene segment can be drawn as well (Figure 5e). Indeed, for structure **4**, resonant forms can be drawn with single radicals on each of the carbon atoms of the same sublattice across the whole molecule (see Figure S4 for selected examples). Nevertheless, it must be noted that resonant forms with the radical on the right hexacene (Figure 5e) display one Clar sextet less than those with the radical on the left hexacene (Figure 5d) and also a π-bond on the four-membered ring. Because resonant forms with the highest possible number of Clar sextets are energetically favored^51^ and the cyclobutene character disfavored with respect to radialene-type structures,^19,52^ the radical may be expected to locate preferentially on the left hexacene. Once again, all of the above is in excellent agreement with the molecular orbital of the singly occupied state (Figure 5f), which extends in this case over the whole molecular structure on carbon atoms of the same sublattice but with a markedly larger amplitude on the left hexacene.

These analyses of structures **3** and **4** therefore reveal one important difference, namely, the larger delocalization of the radical state in **4** compared to **3**. Other parameters remaining unchanged, the higher the delocalization of the electrons the less strongly bound they are to the nuclei, setting the basis for its inverse correlation with the ionization energy. An intuitive picture to understand this is that an increased delocalization implies orbitals made up by linear combinations of a larger number of p_*z*_ electrons, thus filling higher energy (i.e., more weakly bound) states. This correlation has also been observed experimentally comparing similarly sized structures with and without cross-conjugation, the latter reducing the delocalization and therefore increasing the ionization energy.^53−55^ The same is found on closely related oxyradicals differing in their orbital extension.^26^ The energetic shift, however, does not extrapolate to the unoccupied electronic state, as it is counteracted by the stronger Coulomb repulsion U associated with more localized orbitals. All in all, the notably higher ionization energy of **3** — which prevents it from donating an electron to the high work function substrate Au(111), unlike **4**, despite their closely related isomeric structures — is directly related with the lower degree of radical delocalization.

In summary, we have demonstrated a facile synthesis of open-shell acene-derived oxyradicals and the critical effect of the substrate on their magnetic ground state through charge transfer. Systematically comparing closely related chemical structures, we have shown how the energy level alignment of the radical states, which determines the presence or absence of charge transfer between molecule and substrate, depends not only on the number of electron-withdrawing functional groups but also on the delocalization of the electronic state hosting the unpaired electron. This work valuably complements our knowledge on the electronic descriptors determining the magnetic properties of open-shell molecules and on their inter-relations, which is critical for the ultimate design of molecular materials with tailored functionalities.

## Supporting Information Available

Methods description for sample preparation, STM/STS characterization, precursor synthesis, theorerical calculations, supplementary STM images (large scale and for varying preparation conditions), proposed epoxy reaction paths, and complementary resonant structures for products **3** and **4** (PDF)
