# Supplementary material for: Relating Radical Delocalization, Charge Transfer, and Magnetic Ground State in Acene-Derived Oxyradicals
Source: Nano Lett. 2025 Apr 14;25(16):6516–23. doi: 10.1021/acs.nanolett.5c00263 (PMC12023016; doi:10.1021/acs.nanolett.5c00263)
Supplement: Supplementary file 1 — nl5c00263_si_001.pdf [file nl5c00263_si_001.pdf]

Supplementary Information

Relating Radical Delocalization, Charge Transfer and Magnetic Ground State in Acene-Derived Oxyradicals

Tao Wang,[1,2,†,] Sergio Salaverría,[3,†] Fernando Aguilar-Galindo,[4,5,†,*] Javier Besteiro-Sáez,[6] Luis M. Mateo,[6] Paula Angulo-Portugal,[7] Jonathan Rodríguez-Fernández,[3,8] Dolores Pérez,[6] Martina Corso,[2,7] Diego Peña,[3,*] and Dimas G. de Oteyza [2,3,*]*

[1] State Key Laboratory of Organometallic Chemistry, Shanghai Institute of Organic Chemistry, University of Chinese Academy of Sciences, Chinese Academy of Sciences, Shanghai 200032, China

[2] Donostia International Physics Center, 20018 San Sebastián, Spain

[3] Nanomaterials and Nanotechnology Research Center (CINN), CSIC-UNIOVI-PA, 33940 El Entrego, Spain

[4] Departamento de Química, Universidad Autónoma de Madrid, 28049 Madrid, Spain

[5] Institute for Advanced Research in Chemical Sciences, (IAdChem), Universidad Autónoma de Madrid, 28049 Madrid, Spain

[6] Centro Singular de Investigación en Química Biolóxica e Materiais Moleculares (CiQUS) and Departamento de Química Orgánica, Universidade de Santiago de Compostela, Santiago de Compostela, Spain

[7] Centro de Fisica de Materiales (CFM / MPC), CSIC-UPV/EHU, San Sebastián, 20018, Spain

[8] Physics Department, University of Oviedo, 33007 Oviedo, Spain

† These authors contributed equally.

METHODS

Sample preparation and STM/STS characterization

Sample preparation was performed on Au(111) single crystals previously cleaned by repeated cycles of Ar^+ sputtering (1.5 keV) and annealing (500 °C). Precursor molecules **1** were deposited from home-made Knudsen cells at around 220 ˚C. Substrate annealing activated the reactions that lead to the described products. STM measurements were performed using a commercial Scienta-Omicron LT-STM at 4.3 K. The system consists of a preparation chamber with a typical pressure in the low 10^{-10} mbar regime and a STM chamber with a pressure in the 10^{-11} mbar range. To obtain BR-STM images, the tip was functionalized with a CO molecule that was picked up from the Au(111) surfaces. CO was dosed into the STM chamber via a leak valve at a pressure of approximately 1×10^{-8} mbar. For CO adsorption onto the sample, the STM thermal shields were opened and closed again when reaching a sample temperature of 7.0 K. Three such shield opening cycles were typically applied. CO can be picked up with a metallic tip by scanning with a high current and negative bias (e.g. $I = 1$ nA, $U = -0.5$ V). dI/dV spectroscopy measurements were recorded with the internal lock-in of the Nanonis electronics. The oscillation frequency used in experiments is 731 Hz and the amplitude is 2 mV for all scanning tunneling spectroscopies shown in the manuscript.

Precursor synthesis

The synthesis of precursor **1** has been previously described in ref. [1] .

Theoretical calculations

All the calculations have been performed in the framework of the Density Functional Theory. Molecules in the gas phase have been optimized with the Gaussian16 [2] software at the HSE/cc-pVDZ level of theory.[3] Vertical ionization energies have been obtained through single point calculations of their corresponding cations on the neutral optimized structures.

Simulated dI/dV maps were obtained following a methodology similar to the one developed by Jelinek et al.[4,5] with our own code, as in previous works.[6] In these simulations, we have considered a functionalized (CO) tip, which includes s-wave and p-wave orbitals. Molecular orbitals were obtained from gas-phase calculations.

SUPPLEMENTARY FIGURES

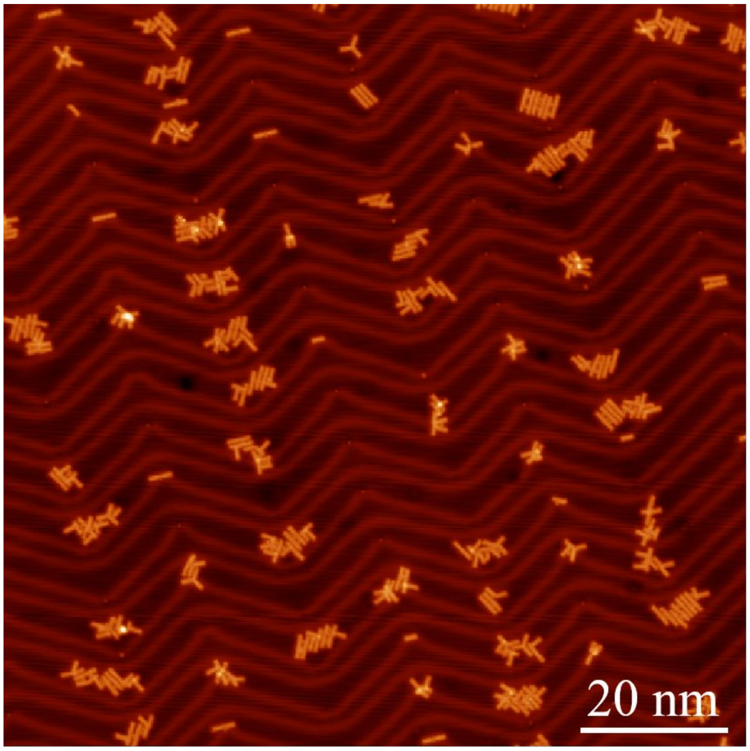

Fig. S1. Large-scale STM image of the sample prepared at 230 °C. More than 70% products are dimers along with a few trimers and tetramers. Scanning tunneling parameters: U= 1 V, I= 100 pA.

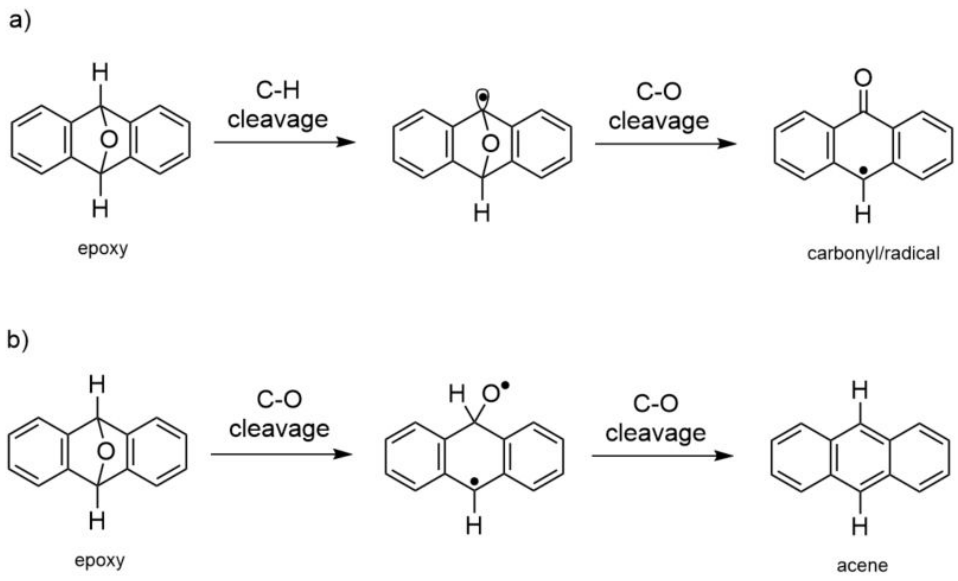

Fig. S2. Proposed reaction paths for (a) the transformation of epoxy into carbonyl groups and (b) for the epoxy dissociation. The pathway (b) is expected to display a higher energy barrier, since it typically occurred during a "hot deposition" annealing procedure, in which sufficient energy was offered just after deposition. In contrast, a slow annealing treatment predominantly followed pathway (a) because it displays lower energy reaction barrier.

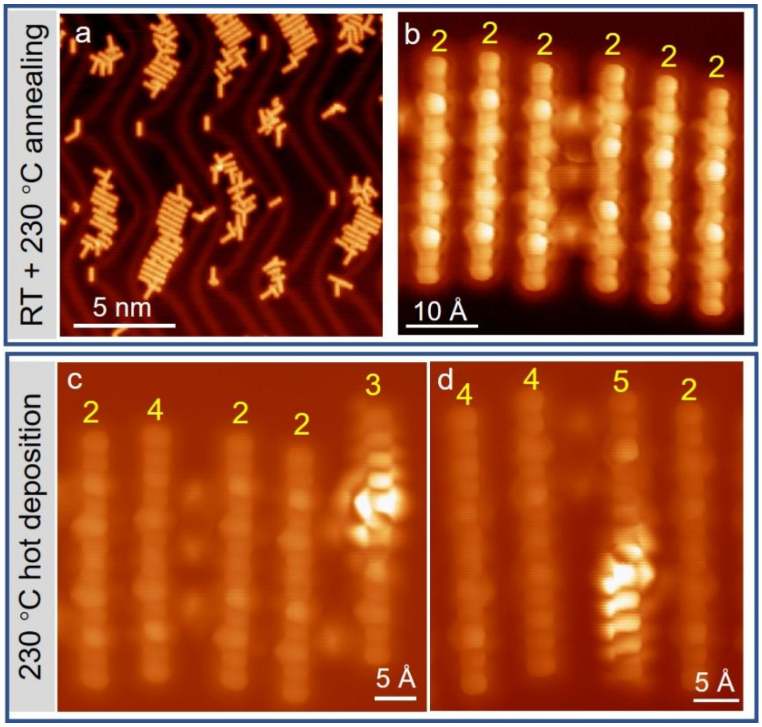

Fig. S3. Comparison of the sample products obtained for different preparations. For the top images, the molecules are deposited on a sample held at RT and annealed afterwards to 230 °C. This renders dimer **2** as the main product. The bottom images are representative for a sample in which the precursors are deposited on a substrate readily held at 230 °C. This facilitates the entire removal of the epoxy groups and thus increases the number of molecules with less than four carbonyl groups. Scanning tunneling parameters: (a) U= 1 V, I= 100 pA; (b-d) U= 5 mV at constant heightmode.

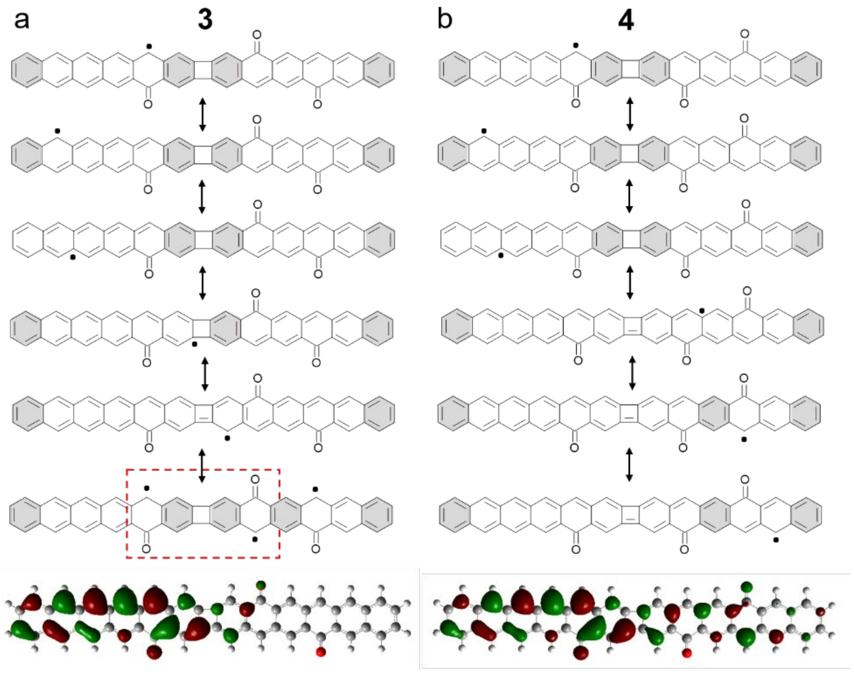

Fig. S4. Resonant structures for molecule **3** (a) and molecule **4** (b) and the singly occupied molecular orbital as calculated by DFT for the molecules in gas phase. Clar sextets are marked with a grey background. Resonant forms with less Clar sextets denote a lower orbital amplitude at the carbon atom hosting the radical.

References

(1) Besteiro-Saez, J.; Mateo, L. M.; Salaverria, S.; Wang, T.; Angulo-Portugal, P.; Calupitan, J. P.; Rodriguez-Fernandez, J.; Garcia-Fuente, A.; Ferrer, J.; Pérez, D.; Corso, M.; De Oteyza, D.; Peña, D. [19]Starphene: Combined In-Solution and On-Surface Synthesis Towards the Largest Starphene. *Angew Chem Int Ed* **2024**, e202411861. https://doi.org/10.1002/anie.202411861.

(2) Frisch, M. J.; Trucks, G. W.; Schlegel, H. B.; Scuseria, G. E.; Robb, M. A.; Cheeseman, J. R.; Scalmani, G.; Barone, V.; Petersson, G. A.; Nakatsuji, H.; Li, X.; Caricato, M.; Marenich, A. V.; Bloino, J.; Janesko, B. G.; Gomperts, R.; Mennucci, B.; Hratchian, H. P.; Ortiz, J. V.; Izmaylov, A. F.; Sonnenberg, J. L.; Williams-Young, D.; Ding, F.; Lipparini, F.; Egidi, F.; Goings, J.; Peng, B.; Petrone, A.; Henderson, T.; Ranasinghe, D.; Zakrzewski, V. G.; Gao, J.; Rega, N.; Zheng, G.; Liang, W.; Hada, M.; Ehara, M.; Toyota, K.; Fukuda, R.; Hasegawa, J.; Ishida, M.; Nakajima, T.; Honda, Y.; Kitao, O.; Nakai, H.; Vreven, T.; Throssell, K.; Montgomery, J. A., Jr.; Peralta, J. E.; Ogliaro, F.; Bearpark, M. J.; Heyd, J. J.; Brothers, E. N.; Kudin, K. N.; Staroverov, V. N.; Keith, T. A.; Kobayashi, R.; Normand, J.; Raghavachari, K.; Rendell, A. P.; Burant, J. C.; Iyengar, S. S.; Tomasi, J.; Cossi, M.; Millam, J. M.; Klene, M.; Adamo, C.; Cammi, R.; Ochterski, J. W.; Martin, R. L.; Morokuma, K.; Farkas, O.; Foresman, J. B.; Fox, D. J. Gaussian˜16 Revision C.01, 2016.

(3) Krukau, A. V.; Vydrov, O. A.; Izmaylov, A. F.; Scuseria, G. E. Influence of the Exchange Screening Parameter on the Performance of Screened Hybrid Functionals. *The Journal of Chemical Physics* **2006**, *125* (22), 224106. https://doi.org/10.1063/1.2404663.

(4) Hapala, P.; Kichin, G.; Wagner, C.; Tautz, F. S.; Temirov, R.; Jelínek, P. Mechanism of High-Resolution STM/AFM Imaging with Functionalized Tips. *Phys. Rev. B* **2014**, *90* (8), 085421. https://doi.org/10.1103/PhysRevB.90.085421.

(5) Hapala, P.; Temirov, R.; Tautz, F. S.; Jelínek, P. Origin of High-Resolution IETS-STM Images of Organic Molecules with Functionalized Tips. *Phys. Rev. Lett.* **2014**, *113* (22), 226101. https://doi.org/10.1103/PhysRevLett.113.226101.

(6) Calupitan, J. P.; Berdonces-Layunta, A.; Aguilar-Galindo, F.; Vilas-Varela, M.; Peña, D.; Casanova, D.; Corso, M.; De Oteyza, D. G.; Wang, T. Emergence of π-Magnetism in Fused Aza-Triangulenes: Symmetry and Charge Transfer Effects. *Nano Lett.* **2023**, *23* (21), 9832–9840. https://doi.org/10.1021/acs.nanolett.3c02586.
